# Optical coherence tomography angiography in uveitis

**DOI:** 10.1186/s12348-019-0190-y

**Published:** 2019-12-23

**Authors:** Paris Tranos, Evdoxia-Maria Karasavvidou, Olga Gkorou, Carlos Pavesio

**Affiliations:** 1 Vitreoretinal & Uveitis Department, Ophthalmica Clinic, Vas.Olgas 196 and Ploutonos, 546 55 Thessaloniki, Greece; 2 Department of Ophthalmology, Hippokrateio General Hospital of Thessaloniki, 49 Konstantinoupoleos Street, 546 42 Thessaloniki, Greece; 3 Uveitis Department, Moorfields Eye Hospital, 162 City Rd, London, EC1V 2PD UK

**Keywords:** Optical coherence tomography angiography, Uveitis, Chorioretinal inflammation, Dye-based angiography, Blood flow

## Abstract

Before the introduction of optical coherence tomography angiography (OCTA) in the early 2000s, dye-based angiography was considered the “gold standard” for the diagnosis and monitoring of ocular inflammation. OCTA is a novel technique, which demonstrates capillary networks based on the amount of light returned from moving blood cells, providing further information on pathophysiological changes in uveitis.

The aim of this review is to describe the basic principles of OCTA and its application to ocular inflammatory disorders. It particularly emphasizes on its contribution not only in the diagnosis and management of the disease but also in the identification of possible complications, comparing it with fundus fluorescein angiography (FFA) and indocyanine green angiography (ICGA). Although the advent of OCTA has remarkably enhanced the assessment of uveitic entities, we highlight the need for further investigation in order to better understand its application to these conditions.

## Introduction

In recent years various imaging modalities have emerged facilitating effective investigation, diagnosis and monitoring of ocular disorders [1]. In 2006, Makita et al. first described optical coherence tomography angiography (OCTA), a novel imaging modality which utilizes the advances in OCT technology to provide new insight in retinal microvascular changes, without the requirement of intravenous dye injection. This innovative technique provides high-resolution angiographic information that can be objectively correlated to OCT anatomic findings [2–4].

Uveitis is associated with a spectrum of pathologic processes including inflammation, vascular occlusion or leakage, local ischemia, and alteration of cellular mediators. Visually debilitating complications such as macular edema and neovascularization among others may potentially occur. Also, some inflammatory lesions may be difficult to differentiate from a vascular lesion. Early identification and monitoring of these changes may be critical in the optimal management of patients with uveitis. Recent studies suggest that the use of OCTA, in conjunction with the other imaging modalities, can be advantageous in patients with ocular inflammation, revealing features which may improve our knowledge on pathophysiology and natural course of the disease and guide decision making for the uveitis specialist [3].

In this review, a comprehensive overview of the principles of OCTA and its application to ocular inflammatory disorders has been performed.

## Dye-based angiography

### Fundus fluorescein angiography

Fundus fluorescein angiography (FFA) is an imaging modality based on the intravenous administration of fluorescein dye [5]. This technique is widely used predominantly for the demonstration of retinal vascular abnormalities, especially leakage, macular or optic disc edema and non-perfusion [6]. However, vascular features of the retina may be underestimated due to the limited resolution of FFA and, as an example, choroidal neovascularization (CNV) may not be detected due to masking from coexisting subretinal hemorrhage [7]. Fluorescein molecular size is also an important issue, as small molecules leak more quickly and prevent detailed view of the vascular structures [8]. Fundus fluorescein angiography can be associated with mild (nausea, vomiting, sneezing, pruritus) or more severe adverse reactions (syncope, local tissue necrosis, thrombophlebitis, local skin eruptions at the site of injection) [9]. Cardiovascular shock, myocardial infarction, laryngeal edema and bronchospasm have also been reported and are important contributors to the mortality rate [10].

### Indocyanine green angiography

Yannuzzi et al. [11] first described indocyanine green angiography (ICGA), which is usually performed to reveal choroidal involvement in uveitis and identify occult choroidal neovascularization (CNV) [12, 13], polypoidal choroidopathy, and CNV complicated with subretinal hemorrhage [14]. In comparison with FFA, the emission wavelength of indocyanine green (ICG) is longer; thus, deeper retinal structures can be better visualized. Additionally, a higher proportion of ICG is albumin bound (~ 98%) [15], so the leakage of dye decreases and the signal to noise ratio improves. Consequently, ICGA is more reliable in imaging the choroidal vasculature and choroidal pathology allowing better visualization of the choriocapillaris and choroidal stroma, which are affected in many visually threatening inflammatory conditions. Nevertheless, this method has limitations similar to FFA since presence of subretinal fluid, subretinal hemorrhage or retinal pigment epithelium (RPE) detachment may also obscure CNV or other retinal features [16]. Severe adverse reactions have also been reported during ICGA, especially in subjects with iodine allergies [15, 17].

## Optical coherence tomography angiography

### Basic principles

Optical coherence tomography angiography is an expansion of spectral-domain optical coherence tomography (SD-OCT) [1], providing detailed visualization of functional vasculature of ocular tissue [18].

The basic principle of OCTA relies on the variation in OCT signal produced by the reflectance of light off the surface of moving red blood cells [1, 18]. Structural tissues generate steady signal, while flowing blood cells produce signal that changes over time because of the continuous motion. OCTA scans are repeated over the same area in order to discriminate the moving particles from static tissue [18]. OCT device emits light which can be reflected, refracted, or absorbed [1] and uses two main methods to develop the angiographic contrast: (1) the amplitude decorrelation (or intensity) and (2) the phase variance. These two methods compare the differences between the light reflected in various vessels and the background, aiming to detect significant motion and allowing detailed depiction of retinal and choroidal microvasculature [1].

### Limitations and artifacts

Despite its many advantages over dye-based angiography techniques, OCTA has some technical limitations. The limited view of field (8 mm x 8 mm) of the currently available devices prevents the wide visualization of the posterior pole and restricts the evaluation of pathology predominantly affecting the peripheral retina. OCTA provides more concise depiction of ocular microcirculation. Nevertheless, it requires accurate segmentation for the identification and quantification of vascular abnormalities of the eye [4].

In line with any other imaging methods, OCTA can be influenced by artifacts which may lead to data misinterpretation.

Media opacities such as cataract, small pupil or defocusing of the light beam have the potential of producing weakened OCT signal and inaccurate flow information. All these shortcomings may be moderated by focusing and centering the OCT beam and by dilating the patients’ pupils [4]. In addition, small eye movements, such as changes of fixation, saccadic or movements associated with tremor and breathing, are sources of artifacts that could lead to overestimation of flow signal [1, 4]. However, eye tracking software and motion correction techniques have become available on commercial OCTA devices, decreasing the defects of OCTA scans [19–21]. Moreover, flowing red blood cells into the vessels of superficial choroidal plexus can cast shadows over the deeper vascular layers [4]. These artifacts result in projection of the same vascular pattern of superficial layers on deeper retinal circulation including the normally avascular layer of photoreceptors. Consequently, detection and quantification of vascular abnormalities in the outer retina may become difficult due to blockage from projection artifacts, which can be misinterpreted [22]. OCTA devices have a threshold for slow flow signal detection; hence, areas with blood flow under this threshold may not be detected and incorrectly maybe characterized as non-perfused [1].

## Optical coherence tomography angiography in uveitis

### Optical coherence tomography angiography vs dye-based angiography

Due to its micromolecular properties, fluorescein may easily leak from retinal vessels with the slightest disruption of the blood-retinal barrier. FFA is a very sensitive imaging modality for the detection of retinal vessel inflammation, because even minor inflammation of the retinal vessel wall may result in vascular leakage. This leakage on FFA is extremely useful feature in order to assess the activity of underlying uveitis, both in terms of intensity and extent. On the other hand, OCTA is not able to detect leakage, but can depict changes in the vessel density of superficial or deep capillary plexus in vasculitis. Kim et al. [2] identified that the parafoveal capillary density in the superficial retinal plexus was significantly lower in eyes with retinal vasculitis compared with healthy ones. These results suggest that OCTA can potentially be used to quantitatively measure the effects of intraocular inflammation.

Dye-based angiography poorly visualizes the deep capillary plexus (DCP) in contrast to OCTA. Evaluation of deeper vascular components can be useful in some uveitis entities including birdshot retinochoroiditis, which has been shown to be associated with decreased flow density in the DCP [23]. Lower flow density is not necessarily associated with non-perfusion. Leakage of plasma outside the vessel wall in retinal vasculitis results in decrease of flow velocity inside the vessel. However, in active inflammation accompanied by leakage on FFA, retinal circulation can be identified on OCTA scans despite the lower flow velocity.

Dye leakage in posterior uveitis may be helpful for the assessment of the inflammatory activity, but it can be restrictive in the evaluation of adjacent capillary perfusion. In this respect, the use of OCTA has proven advantageous, providing details on microvascular morphology and information about capillary perfusion in both superficial and deep capillary plexus.

FFA is considered the “gold standard” for the detection of any type of CNV [24]. However, even with multimodal imaging, it is very difficult to differentiate active inflammatory lesions from inflammatory CNV, as both have the potential to cause a breakdown in the blood-retina barrier. Usually, inflammatory CNV is characterized by early hyperfluorescence with late leakage, while inflammation shows early hypofluorescence with late hyperfluorescence on FFA [25]. Not infrequently, this early hyperfluorescence of CNV may not be obvious due to blockage from inflammatory component or hemorrhage. Moreover, inflammatory lesions may show early hyperfluorescence because of the window defect caused by RPE damage, which may be present adjacent to new active lesions.

ICGA provides better imaging of the choroidal vasculature through the RPE [26] since in contrast to FFA, the lesions are not obstructed by significant choriocapillaris leakage [27]. ICGA leakage results from significant damage to retinal and choroidal stroma vessels, but dye leakage from fenestrated choriocapillaris may be very slow in case of inflammation. Herbort et al. demonstrated the involvement of choriocapillaris and choroidal stroma in the pathophysiology of several inflammatory diseases of the fundus which can cause characteristic findings on ICGA [28].

### Microvascular changes in uveitis

OCTA is the only imaging method that can depict microvascular changes of the superficial and deep capillary plexus in detail, since there is no masking by leakage, pooling or window defects [29, 30] (Table 1).

**Table 1:** Pathological features detected on different layers of OCTA in various uveitis entities

	Superficial capillary plexus	Deep capillary plexus	Outer retina	Choriocapillaris	Choroid
Retinal vasculitis	-Decreased flow density -Enlargement/ Irregularity of FAZ -Capillary remodeling	-Grayish hypo/non perfused areas -Elevated, dilated or shunting perifoveal vessels -Well delineated flow void areas			
Birdshot retinochoroiditis	-Telangiectatic vessels -Increased intercapillary space in the perifoveal region				
Ocular toxoplasmosis		Neovascular network arising from retinal vasculature with no contribution of the choroid			
MEWDS				Normal flow	
APMPPE				Flow reduction and ischemia	Flow reduction and ischemia
PIC/MCP			Tangled vessels arising from choriocapillaris	-Focal flow reduction (MCP) -Tangled vessels extending into outer retina	
Serpiginous choroiditis				Flow reduction in areas of active lesions	Better delineated vessels in areas of inactive lesions
Sarcoidosis					Flow void areas
Tuberculosis					-Flow void areas -Tangled vessels arising from choriocapillaris
VKH				Focal flow void	
Uveitic macular edema		Decreased capillary density			
Inflammatory CNV			Neovascular network		

The stability of ocular function at the level of neurosensory retina is largely dependent on the integrity of the inner and outer blood-retinal barrier [31, 32]. The human immune system produces inflammation by releasing various inflammatory mediators including prostaglandins, interleukins (e.g., IL-1, IL-2, and IL-6), interferon gamma and tumor necrosis factor alpha (TNF-a). All these mediators contribute to the disruption of the inner blood-retinal barrier, resulting in an influx of fluid from vessels to the intraretinal or subretinal space and formation of extracellular edema.

Recent studies using OCTA showed that uveitic macular edema is associated with changes in the density or morphology of deep capillary plexus (DCP). Their analysis revealed a significantly lower vessel density in DCP in uveitic eyes complicated by macular edema. In addition, ocular inflammation was associated with parafoveal capillary loss in the superficial capillary plexus regardless of the presence of macular edema [2]. Besette et al. [1] identified similar findings including the remodeling of capillaries and the irregularity and/or enlargement of foveal avascular zone (FAZ) in uveitis patients.

Blood flow changes associated with active inflammation are characterized by capillary dropout or loss in the SCP that can be identified as hypo-perfused areas in OCTA. However, apparent areas of non-perfusion may only represent slow flow, considering the inadequacy of the device in detecting slow flow signal [1].

### OCTA in anterior uveitis

Pichi et al. was the first to demonstrate quantitatively the iris vasculature of subjects with anterior uveitis using OCTA. By applying the appropriate settings for iris scanning, they found that inflamed irises had better highlighted microvasculature than the healthy ones, with radial small vessels packed more densely towards the pupillary margin and irregular vessels arranged less densely towards the iris root [1].

In order to quantify the increase in flow and the dilation of vessels which occur in anterior uveitis, Pichi et al. compared the brightness of grayscale OCTA scans before and after uveitis treatment. Their measurements showed that the brightness was significantly increased in severe inflammation than in mild cases. In addition to measuring brightness, they attempted to calculate the vascular volume of inflamed irises. Study data revealed much higher vascular volume in eyes with 4+ anterior chamber cells, decreasing with improvement of inflammation. Those results based on OCTA measurements established a new perspective in the qualitative and quantitative assessment of iris vasculature using OCTA. However, this approach is limited due to some anatomical and technical parameters:

1. Pupil size needs to be constant as it may affect the morphology of iris vasculature and OCTA results.
2. OCTA measurements may not be reproducible because of differences in iris pigmentation.
3. Ocular tissue refraction of OCTA beam can give a false impression of anterior segment physical characteristics.

### OCTA in retinal vasculitis

Retinal vasculitis may be a consequence of various ocular and systemic diseases including systemic lupus erythematosus (SLE), Adamantiades-Behçet disease (ABD), multiple sclerosis (MS), ocular tuberculosis (TB), and sarcoidosis among others. Inflammation of retinal vessels may be a component of almost all types of intermediate and posterior uveitis.

In active vasculitis, fundoscopy reveals focal, multifocal, or diffuse white sheathing of retinal vessels. Perivascular infiltration by inflammatory cells accounts for the pathological vessel sheathing, which is postulated to generate the blurred margins of vessels. Retinal vasculitis may be accompanied by other vascular changes including telangectasias, vascular anastomosis, microaneurysms, macroaneurysms and optic disc or preretinal neovascularization [1].

FFA remains a remarkably useful imaging modality for the diagnosis and monitoring of retinal vasculitis. Fluorescein is able to escape through breaks in blood-retinal barrier, allowing the detection of vascular occlusion or leakage [3]. On the other hand, recent studies have shown that OCTA can demonstrate enlargement and/or irregularity of the foveal avascular zone (FAZ) and capillary remodeling [1], specifically in patients with involvement of the macula.

Khairallah et al. [33] with the aid of OCTA highlighted the presence of grayish nonperfused/hypoperfused areas mostly involving the deep capillary plexus (DCP) in patients with ABD vasculitis. Similar findings have been observed in retinal vascular occlusion, diabetic retinopathy and sickle cell retinopathy [34–38]. Deep capillaries are more susceptible to ischemia as they are not directly connected to arterioles. Additional findings included elevated, dilated, or shunting perifoveal capillary vessels and well-delineated black, roundish, or elongated areas devoid of flow. The formation of cystoid spaces displacing the peripheral capillaries might explain the total absence of flow in these areas. However, caution should be taken as retinal atrophic alterations, involving the macula and the retinal nerve fibers, may produce projection artifacts on OCTA, indicating nonperfusion/hypoperfusion of both the superficial and deep capillary plexus, where none exists.

### OCTA in retinitis and choroiditis

#### Birdshot retinochoroiditis

Birdshot retinochoroiditis (BRC) is a rare bilateral chronic posterior uveitis, strongly associated with human leukocyte antigen (HLA) A29 [39]. Clinical picture is characteristic, with multiple scatter, oval, creamy white hypopigmented choroidal lesions, vitritis, retinal vasculitis, and macular edema.

De Carlo et al. [40] performed OCTA on eight eyes with BRC to evaluate the microvascular alterations occurring in the superficial retinal capillary plexus of the posterior pole. Their study data revealed the presence of abnormal telangiectatic vessels and an increase of intercapillary spaces in all affected eyes. They also observed capillary dilatations and loops in seven of the total eight eyes examined.

Twenty-two patients (forty-four eyes) with BRC, which were considered inactive based on clinical examination and imaging with OCT and FFA, were evaluated by Pichi et al. [1]. In their series, foveal avascular zone (FAZ) and the area of capillary non-perfusion were delineated and measured with the use of OCTA at the level of superficial capillary plexus (SCP). Their results showed that subjects with BRC had a larger FAZ area compared with the healthy ones. Another important microvascular change being noticed was the enlargement of perifoveal intercapillary spaces in all eyes with BRC, representing areas of perifoveal ischemia. All these findings could lead to the assumption that chronic BRC induces hypoperfusion of the SCP, resulting in tissue hypoxia and cellular death.

In another study held by Pohlmann et al. 64 eyes of 32 subjects with BRC were classified according to disease activity and duration of the disease. They were evaluated with multimodal imaging which revealed that active BRC was associated with retinal vasculitis and hyperfluorescence of the optic disc on FFA as well as hypofluorescent areas on ICGA. In all eyes, OCTA demonstrated capillary loops and telangiectatic vessels, altered retinal vascular architecture and rarefication of C-scans in retinal layers. However, increased rarefication of C-scans and altered retinal vascular architecture in SCP and DCP were significantly correlated with disease activity [41].

#### Ocular toxoplasmosis

Ocular toxoplasmosis is frequently associated with retinal vasculitis, and may also result in serous retinal detachment, occlusive vascular disease, macular edema and CNV. Dye-based angiography is usually performed for the diagnosis of atypical cases and the identification of complications. On FFA, the lesion appears hypofluorescent in the early images followed by gradual leakage from surrounding vessels. In the presence of CNV, a typical progressive leakage over the area of new vessels is demonstrated on FFA. On ICGA, the main lesion of ocular toxoplasmosis is depicted as a hypofluorescent area with multiple satellite hypofluorescent foci, whereas CNV appears hyperfluorescent [3]. ICGA also shows involvement of the choriocapillary and choroid in acute toxoplasmic retinitis.

Spaide [42] recently described the use of OCTA in order to evaluate an area of CNV in a patient with ocular toxoplasmosis. Leakage on FFA indicated the presence of CNV but its precise anatomy could not be identified. OCTA scans showed significant contribution to the neovascular tissue from the retinal vasculature without any participation from the choroid. Hence, it is obvious that we can perform OCTA to assess the origin of abnormal vessels developed not only in toxoplasmosis but also in other inflammatory diseases.

### OCT-A in choroiditis (choriocapillaropathies and stromal choroiditis)

#### Multiple evanescent white dot syndrome

Multimodal imaging can be extremely useful in patients with multiple evanescent white dot syndrome (MEWDS) and subtle clinical findings. Electroretinography (ERG) demonstrates photoreceptor dysfunction [43], while in fundus autofluorescence (FAF) hyperfluorescent spots are highlighted in the early stages, as the RPE autofluorescence is unmasked due to misalignment of the photoreceptors or metabolic changes affecting RPE [44]. FFA shows early hyperfluorescence of the dots with late staining, while on ICGA more numerous hypofluorescent spots are illustrated than the corresponding seen clinically or on FFA [45, 46]. On structural en face OCT hyporeflectivity is seen at the level of the RPE-photoreceptor complex that co-localizes with the hypofluorescent spots on ICGA, supporting the hypothesis that the dark areas on ICGA are attributable to focal non-functional RPE cells not absorbing the ICG molecule. OCTA is of great importance in revealing that within the corresponding hypofluorescent spots of ICGA, the choriocapillaris and retinal capillary blood flow are completely normal. This reinforces the concept that choriocapillaris may not be involved in this disease and the primary cause of it stands at the level of RPE-photoreceptor complex [1, 23, 47–49]

#### Acute posterior multifocal placoid pigment epitheliopathy

FFA demonstrates early hypofluorescence of the acute posterior multifocal placoid pigment epitheliopathy (APMPPE) lesions and late hyperfluorescence due to staining [50–52]. APMPPE has been proposed to be a result of ischemia and acute inflammation of the choriocapillaris, but not of the medium and large sized choroidal vessels, leading to RPE abnormalities [53–55]. This inner choroidal involvement as prominent feature is supported by the ICGA findings of hypofluorescent areas corresponding to the lesions observed on FFA [51, 52, 56, 57]. OCT imaging manifests increased inner choroidal hyporeflectivity or lucency [52, 58], ellipsoid zone disruption and sub-RPE drusenoid abnormalities with RPE atrophy ensuing in the course of the disease, all of which co-localize with the primary zone of choroidal hypoperfusion [1, 59]. OCTA imaging confirmed that hypothesis of inner choroidal or choriocapillaris flow reduction, as it illustrates true choriocapillaris flow-void areas that correspond to the ICGA hypofluorescent spots and are of the same or larger size. These findings are not artifacts, since they are not associated with signal attenuation or blockage due to overlying RPE alterations [1, 23, 58–63]. Other studies have also showed progressive recovery of blood flow with reduction of the ill perfused area and evidence of vascular reperfusion [1, 59, 60, 62].

#### Multifocal choroiditis and panuveitis

Along with direct damage of the retina and RPE due to inflammation, common vision-threatening complications of multifocal choroiditis and panuveitis (MCP) include inflammatory CNV, cystoid macular edema and subretinal fibrosis [64–67]. In active disease, FFA shows early hypofluorescence due to blockage from the lesions and late hyperfluorescence due to staining, while atrophic scars look hyperfluorescent due to window defect [3, 68, 69]. ICGA illustrates hypofluorescent acute lesions, some of which may not be clinically apparent [26]. On OCT inflammatory lesions are typically presented as homogenous elevations of the RPE that may penetrate into the subretinal space and outer retina [1, 23, 66, 70, 71]. More heterogeneous subretinal material, occasionally with a sub-RPE component, may indicate the presence of CNV [23, 66, 71, 72]. However, differentiating purely inflammatory lesions from inflammatory CNVs can be potentially difficult, as both lesions may leak on FFA [23, 66]. OCTA has been used to assess vascular alterations and is able to precisely identify CNV as a lacy vascular network at the level of a normally avascular area of outer retina. Although OCTA cannot accurately define the level of CNV activity in cases of inflammatory CNV, it is the diagnostic modality of choice since findings of other imaging modalities can be largely equivocal and difficult to interpret [1, 3, 23, 64, 66, 73, 74]. Therefore, OCTA findings are crucial and may entirely determine the therapeutic approach, because a combination of immunosuppressive agents with intravitreal anti-VEGF therapy in cases of inflammatory CNV is mandatory, as opposed to monotherapy with immunosuppression in purely inflammatory lesions without CNV. OCTA at the level of choriocapillaris has also shown flow-void areas that correspond to the hypofluorescent areas on ICGA. However, caution should be taken in cases where RPE atrophy is present, which may result in a hyper intense vascular network on OCTA, attributable to the visible Sattler layer’s vessels under the atrophic RPE and the undetected signal of the choriocapillaris blood flow [73].

#### Punctate inner choroidopathy

The clinical course of punctate inner choroidopathy (PIC) is considered to be benign unless complicated with vision-threatening consequences, which include subretinal fibrosis and CNV [3, 66, 75, 76].

**Fig. 1:**
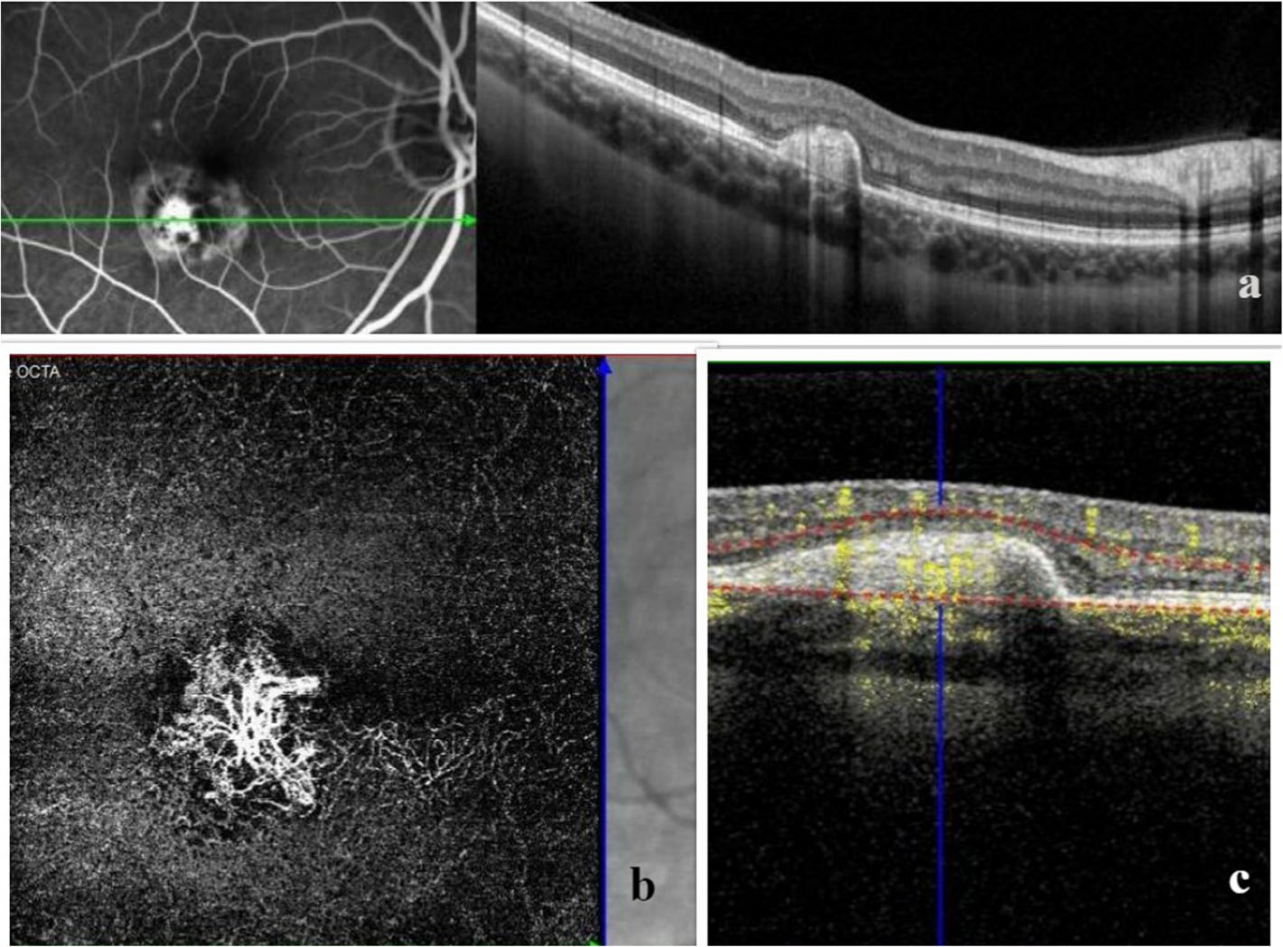
FFA and structural OCT of a 36-year-old myopic woman with punctate inner choroidopathy complicated by choroidal neovascular membrane (CNV) (**a**). OCTA illustrates the lacy pattern of the CNV (**b**) associated with blood flow (yellow color) within the fibrovascular pigment epithelial detachment in the combined structural OCT/OCT-A (**c**)

In the acute phase, FFA depicts early hypofluorescent spots with late staining, while CNV is typically illustrated with a lacy hyperfluorescent pattern followed by late leakage [3, 77, 78]. On ICGA hypofluorescent spots are evident at the level of choriocapillaris [3]. On OCT active lesions are typically presented as elevations of the RPE, which may extend into inner retina, which decrease converting to areas of sub-RPE accumulations or outer retinal disruption when inactive. Hyperreflective heterogeneous subretinal material, accompanied or not with intraretinal fluid, may be indicative of a neovascular membrane [3, 23, 66]. OCTA has been shown to effectively reveal and confirm the presence of a neovascular network of choroidal origin in patients with PIC, even in apparently inactive cases, suggesting an increased risk of recurrence [79] (Fig. 1).

Similar to MCP, OCTA findings can be advantageous in differentiating purely inflammatory lesions from inflammatory CNV, in cases where findings from other imaging modalities are inconclusive. Moreover, it can be useful in monitoring patients with CNV and its response to therapy [3, 66].

#### Serpiginous choroiditis

**Fig. 2:**
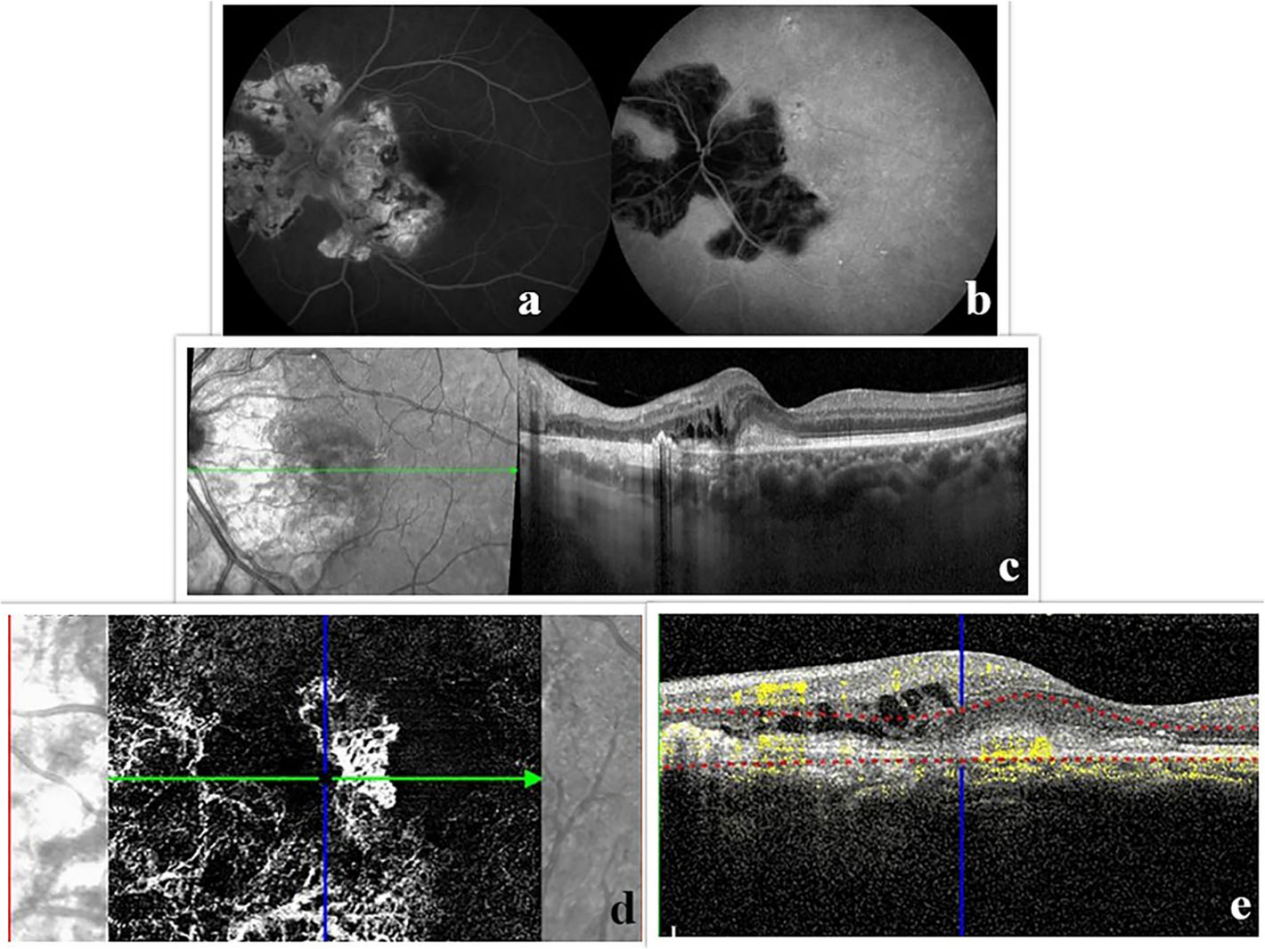
Multimodal imaging of a 56-year-old Caucasian man with serpiginous chorioretinopathy. Late frame of combined FFA and ICG illustrating staining and hypofluorescence of the placoid lesion respectively (**a**, **b**), OCT demonstrates the presence of a hyper-reflective subfoveal lesion accompanied by accumulation of intraretinal fluid and disorganization of the outer retina nasal to the lesion (**c**). OCTA reveals the presence of a type II choroidal neovascular membrane associated with blood flow in the combined structural OCT/OCT-A (**d**, **e**). Note the blood flow at the nasal aspect of the same slab corresponding to projection artifact of the normal overlying retinal vessels

Fundus autofluorescence (FAF) is remarkably useful in evaluating the inflammatory activity of serpiginous choroiditis and the risk of its progression [80]. On FAF inactive lesions are shown hypofluorescent with sharp borders, while active lesions appear as areas of hyperfluorescence at the margin of the hypofluorescent lesions [81]. On FFA active lesions show early hypofluorescence with late hyperfluorescence and inactive ones show window defects. ICGA of inactive lesions reveals marked hypofluorescence throughout all phases of the angiogram (Fig. 2a, b). On both FFA/ICGA perfusion defects of the choriocapillaris seem to be more extensive than the RPE damage shown on FAF, which suggests that choroidal damage precedes RPE damage [80, 82]. OCTA of choriocapillaris on active lesions shows clearly demarcated flow-deficit areas that correspond precisely to the hypofluorescent lesions on ICGA [82, 83]. Conversely, inactive lesions demonstrate some detectable flow within the areas of flow void, indicating deeper medium-to-large choroidal vessels existence and choriocapillaris loss, better detected on OCTA than on ICGA [23, 84, 85].

#### Sarcoidosis

In sarcoidosis, FFA is useful in revealing retinal vasculitis, while ICGA illustrates punctuate hypofluorescence [26]. OCTA may miss small granulomas with diameter smaller than the size of choriocapillaris lobules. In contrast, larger granulomas which occupy the full thickness of the choroid [86, 87], block the choriocapillaris, leading to the appearance of flow void areas on OCTA [1]. These areas co-localize with the hypofluorescent spots on ICGA, as well as with the hyporeflective choroidal lesions on enhanced depth imaging OCT (EDI-OCT) [1, 23].

#### Tuberculosis

Since tuberculosis (TB) is predominantly a choroidal disease, ICGA provides useful imaging information in patients with TB posterior uveitis effectively detecting even very subtle lesions, not visible on FFA and FAF [88–90]. Partial thickness tubercular choroidal granulomas are shown as hypofluorescent lesions that become isofluorescent during the late phases, whereas full thickness ones remain hypofluorescent throughout the study [88, 91, 92]. These granulomas appear as hyporeflective lesions on EDI-OCT [89, 91]. FFA of choroidal tubercles and granulomas shows early hypofluorescence with late hyperfluorescence or isofluorescence as the deep choroidal lesions, may disappear after the early phase of the FFA due to the choriocapillary flush.

Inactive healed tubercles show transmission hyperfluorescence, while large solitary granulomas show early and progressively increasing hyperfluorescence and late pooling of dye in subretinal space of overlying exudative retinal detachment [93–95].

OCTA is able to depict these lesions as well-delineated flow void areas due to hypoperfusion of the choriocapillaris, correlated well with the findings on ICGA and EDI-OCT. Few preserved islands of choriocapillaris may appear in the center of these areas. During the healing process, as a result of the choriocapillaris atrophy in some patients, medium-to-large choroidal vessels may be visualized in choriocapillaris zone on en face OCTA [89]. Vascular abnormalities, including non-neovascular tufts, tangled vessels and neovascular membranes may also be assessed with OCTA, which clearly demarcates the involvement of various retinochoroidal layers [85, 89].

#### Vogt-Koyanagi-Harada

Multimodal imaging is mandatory in order to differentiate Vogt-Koyanagi-Harada (VKH) from other clinical entities with overlapping features. OCT typically demonstrates multilobular serous macular detachments, with occasional subretinal hyper-reflective material within subretinal fluid that probably represents fibrin. In addition, using EDI-OCT Maruko et al. found increased choroidal thickness during the acute phase. FFA shows multifocal hyperfluorescent dots at the level of the RPE due to leakage or staining and late central pooling of dye in subretinal space. ICGA shows evenly distributed hypofluorescent spots (indicating active inflammation of the choroid) which may remain hypofluorescent or become isofluorescent during the late phase, depending on the depth of choroidal involvement. On OCTA, multiple dark foci are illustrated in the choriocapillaris layer, with the slab located below the RPE-Bruch’s membrane complex [1, 96] indicating loss or severe hypoperfusion of the choriocapillaris. These foci appear as areas of flow void with discrete and sharply demarcated edges that consistently overlap with the hypofluorescent spots of the ICGA [23, 97]. This, in conjunction with no signal loss on the structural en face OCT, supports the hypothesis of true focal choriocapillaris ischemia [98, 99].

## Complications of uveitis

### Macular edema

Spectral-domain OCT (SD-OCT) is the imaging modality of choice for the identification and monitoring of uveitic CME as well as the evaluation of its response to treatment [100, 101].

**Fig. 3:**
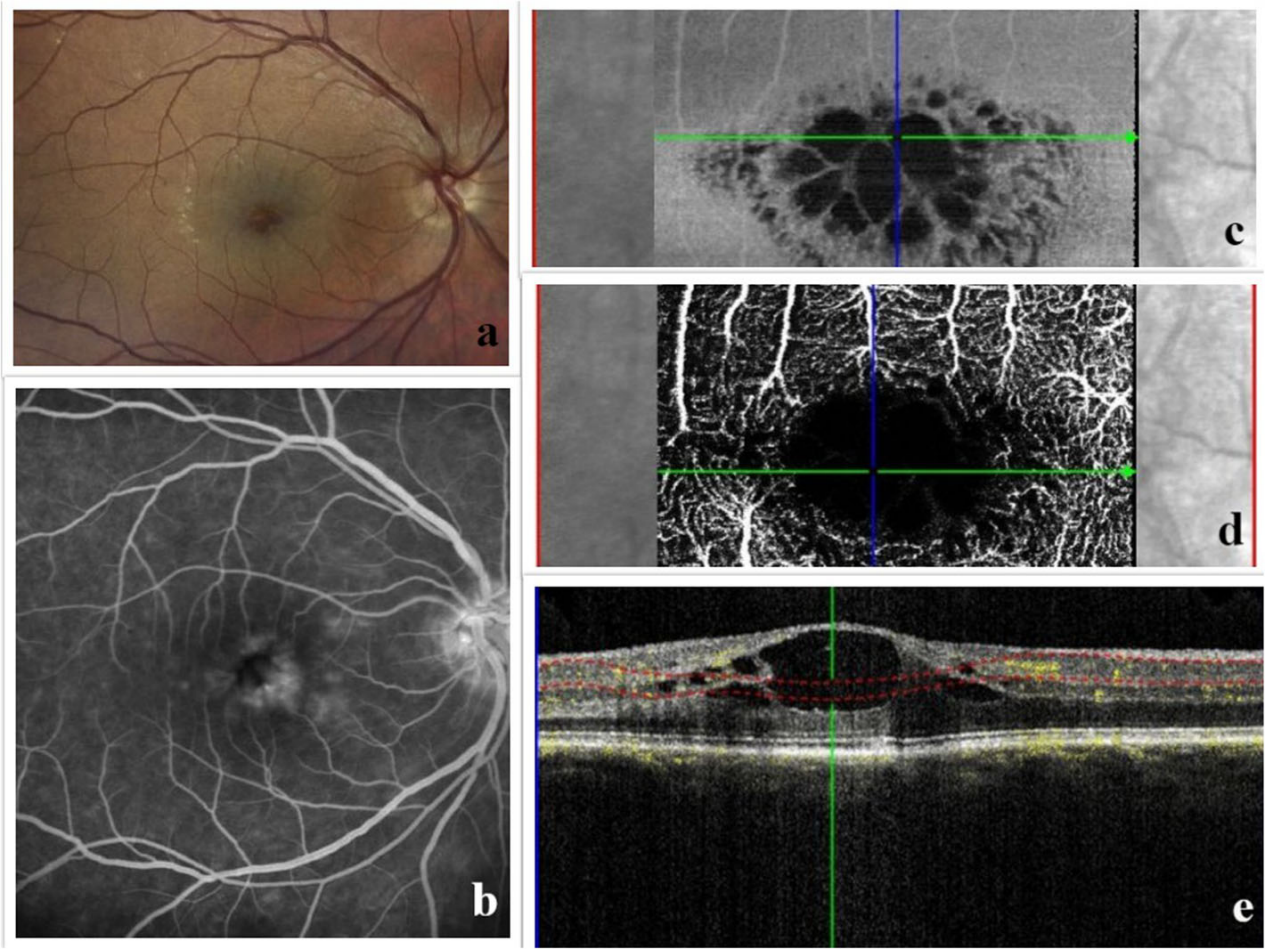
Multimodal imaging of a 36-year-old Caucasian woman with intermediate uveitis. Color photo showing cystoid spaces with abnormal foveal reflex (**a**), FFA demonstrating petaloid pattern of fluorescein leakage along with hyperfluorescence of the optic disc (**b**), C-scan taken at the level of superficial vascular plexus with the corresponding en-face image exhibiting clearly visible cystoid lesions (**c**), the OCT-A illustrates flow void cystoid areas at the macula coupled with an enlargement of the foveal avascular zone (**d**), the B-scan OCT angiogram passing through the foveal depression (**e**)

Carrying out a quantitative OCTA analysis, Kim et al. [2] highlighted the presence of distinct areas with impaired retinal perfusion in patients with uveitic macular edema. More specifically, their data revealed remarkably decreased vascular density parameters, including skeleton and vessel density in deep retinal layer (DRL) of uveitic eyes with active CME compared with normal eyes. These alterations in deep capillary plexus (DCP) corresponded to the site of intraretinal cystoid spaces in inner retina, suggesting that CME may lead to displacement of retinal capillaries or even attenuation of the DRL signal (Fig. 3).

### Retinal ischemia

Uveitis is frequently associated with vascular inflammation, which may lead to occlusive vasculitis characterized by retinal ischemia. Fundoscopy reveals cotton-wool spots and hemorrhages, followed by the development of telangiectatic vessels and neovascularization in more severe cases [102].

Retinal vascular occlusion is demonstrated on FFA as areas of capillary shutdown. OCTA may facilitate the evaluation of such patients by providing detailed information on ischemic areas, which present with increased intercapillary spaces on OCTA scans. The limitation, as previously mentioned, is the inability to obtain OCTA images of the peripheral retina.

### Retinal neovascularization

Retinal NV may develop in uveitic patients through inflammatory and ischemic mechanisms. It is frequently seen in Behçet’s disease (ABD), sarcoidosis, pars planitis, Eales disease, tuberculosis, systemic lupus erythematosus (SLE), and idiopathic retinal vasculitis [103, 104].

Although FFA is capable of demonstrating retinal NV, it cannot separately illustrate the major retinal capillary networks (superficial and deep) and may fail to reveal retinal ischemia in some of the aforementioned diseases [105]. OCTA is the imaging modality that provides detailed delineation of the microvascular structures and their potential abnormalities [105–107]. Furthermore, OCTA facilitates assessment of the precise spatial extent (depth and size) and morphological features of these intraretinal abnormalities that cannot be clearly visualized on FFA [106, 108]. Neovascular complex usually appears on OCTA as a small tuft of high-flow tiny vessels with curvilinear morphology, while abnormal retinal circulation’s communications or pathological retinal-retinal anastomosis can also be detected [106, 109–111]. OCTA may also be sensitive enough to reveal the extent of macular ischemia and record vascular flow changes during the course of the disease [106, 112]. However, vascular alterations in far or mid-periphery have to be determined by FFA [106].

### Choroidal neovascularization

Choroidal neovascularization (CNV) is one of the main sight-threatening complications in uveitis with an incidence of 2% within uveitic population [113]. It predominantly occurs in eyes with posterior uveitis, being particularly common in MCP, PIC, ocular histoplasmosis syndrome, and serpiginous choroiditis [3, 64–66, 75, 78, 79, 114, 115] (Fig. 2c).

It has been suggested that a combination of inflammatory and associated ischemic events decompensate the previously established balance between normal angiogenetic factors and various inflammatory mediators. The choroid-produced vascular endothelial factor (VEGF) and inflammatory mediators, including interleukin-1 (IL-1) and tumor necrosis factor a (TNF-a), play a role in the development of inflammatory CNV through the disruption of the choriocapillaris, Bruch’s membrane and retinal pigment epithelium (RPE) [1, 3, 116–119].

Early diagnosis of CNV is crucial in order to accomplish more effective management and favorable prognosis of the disease. However, differentiating purely inflammatory lesions from inflammatory CNV can be challenging in uveitic patients [1, 64, 120, 121].

OCTA may facilitate early detection of CNV, when findings of other imaging modalities are inconclusive. It has the utility of delineating a well-circumscribed neovascular network inside the area of a lesion without being obscured by dye leakage [64, 66] (Fig. 2d, e). OCTA is effective in revealing CNV despite existing subretinal fluid or hemorrhage, prevailing in such cases over FFA [64]. In addition, it is very efficient in monitoring the progress of CNV lesions that were under treatment and their response to it. Lesions that show no blood flow signal on OCTA can be distinguished from potential CNV lesions and therefore are classified as purely inflammatory one s[59]. Although it has demonstrated higher sensitivity in identifying CNV than conventional dye-based angiography, it still lacks the ability to determine which neovascular membranes are clinically active [66, 122]. It can be speculated that CNV which are identified on OCTA without active leakage on FFA may represent “quiescent CNV” which requires a different follow-up schedule [64]. S-OCT of the OCTA has lately been proved really helpful in this direction. It allows active flow detection (which is usually colored) and, therefore, valuable information about perfusion of vessels in order to distinguish active CNV lesions [123]. Overall, OCTA alone cannot distinguish between active and inactive CNVs and should be integrated into a multimodal imaging approach [124]. However, it is a critical adjunct in identifying the presence of CNV and, therefore, a co-guide in the therapeutic management and monitoring of those patients [1, 66].

## Conclusions

Undoubtedly OCTA is gaining increasing popularity among retina specialists turning into an invaluable asset in the assessment of various retinal diseases, including uveitis. It has the potential to significantly alter the approach in diagnosis and management of uveitic entities by shedding new light into the pathophysiology of abnormal vascular changes in inflammatory conditions. Therefore, a future of a more optimal, non-invasive and valid monitoring of the uveitic patients is becoming apparent with the contribution of this imaging modality.

OCTA is able to detect and investigate a lesion and its precise spatial extent and to calculate the area and density of microvascular flow, as well as flow void areas, in a three-dimensional manner. Furthermore, it overcomes the burden of the masking effect by other elements noticed in conventional angiography. This along with the potentially future established measurements of other flow indices on OCTA, through the chorioretinal vascular plexus and through the avascular compartments, make OCTA unique in quantifying vascular perfusion and ischemia in uveitic disease. Additionally, earlier detection of a uveitic entity or its recurrence may be possible, even before the manifestation of obvious morphological changes and clinical signs.

OCTA is useful for detecting CNV with some limitations. This imaging modality lacks the ability to delineate activity of neovascular membranes. Certain areas including prevention and identification of image artifacts and projection errors require improvement.

More precise information about deeper structures may also be obtained by future use of longer wavelength monochromatic light, while the requirement of larger fields of view with higher resolution and decreased motion artifacts may be fulfilled by larger scan-patterns or faster scanning speeds. OCTA has the following limitations: inability to illustrate leakage and detect blood flow below the slowest detectable flow. Nevertheless, its non-invasive and reproducible nature can assist in a better patient compliance.

Overall, OCTA is a promising tool that continues to evolve and may diminish the need of dye-based angiograms in the future. It has been shown to have high potential to impact clinical care in uveitis however, despite its promising future, it is of note that there is limited experience in this technology, and further large scale studies are required in order to establish it as an irreplaceable clinical utility.

## Data Availability

Since this is a review article, there are no data repositories for this manuscript.

## Abbreviations

ABD: Adamantiades-Behçet disease
APMPPE: Acute posterior multifocal placoid pigment epitheliopathy
BRC: Birdshot retinochoroiditis
CME: Cystoid macular edema
CNV: Choroidal neovascularization
DCP: Deep capillary plexus
DRL: Deep retinal layer
EDI: Enhanced depth imaging
FAF: Fundus autofluorescence
FAZ: Foveal avascular zone
FFA: Fundus fluorescein angiography
HLA: Human leukocyte antigen
ICGA: Indocyanine green angiography
IL: Interleukin
MCP: Multifocal choroiditis and panuveitis
MEWDS: Multiple evanescent white dot syndrome
MS: Multiple sclerosis
OCT: Optical coherence tomography
OCTA: Optical coherence tomography angiography
PIC: Punctate inner choroidopathy
RPE: Retinal pigment epithelium
SCP: Superficial capillary plexus
SD: Spectral domain
SLE: Systemic lupus erythematosus
TB: Tuberculosis
TB: Tuberculosis
TNF: Tumor necrosis factor
VKH: Vogt-Koyanagi-Harada
